# An Improved Load Forecasting Method Based on the Transfer Learning Structure under Cyber-Threat Condition

**DOI:** 10.1155/2022/1696663

**Published:** 2022-08-24

**Authors:** Luo Zhao, Xinan Zhang, Yifu Chen, Xiuyan Peng, Yankai Cao

**Affiliations:** ^1^College of Intelligent Systems Science and Engineering, Harbin Engineering University, Harbin 150001, China; ^2^Department of Electrical, Electronic and Computer Engineering, The University of Western Australia, Perth 6009, Australia; ^3^Department of Chemical and Biological Engineering, University of Wisconsin-Madison, Madison 53706, USA; ^4^Department of Chemical and Biological Engineering, University of British Columbia, Vancouver V6T 1Z3, Canada

## Abstract

Smart grid is regarded as an evolutionary regime of existing power grids. It integrates artificial intelligence and communication technologies to fundamentally improve the efficiency and reliability of power systems. One serious challenge for the smart grid is its vulnerability to cyber threats. In the event of a cyber attack, grid data may be missing; subsequently, load forecast and power planning that rely on these data cannot be processed by generation centers. To address this issue, this paper proposes a transfer learning-based framework for smart grid scheduling that is less reliant on local data while capable of delivering schedules with low operating cost. Specifically, the proposed framework contains (1) a power forecasting model based on transfer learning which can provide high quality load prediction with limited training data, (2) a novel adaptive time series prediction method with modeling time series from a covariate shift perspective that aims to train the forecasting model with a strong generalization capability, and (3) a day-ahead optimal economic power scheduling model considering a shared energy storage station.

## 1. Introduction

In recent years, the emergence of renewables and big data has prompted a reform of the electrical network. As a consequence, the concept of a smart grid becomes increasingly popular [1]. A smart grid is defined as the next generation electrical grid with power-flow control, self-healing, and energy reliability using digital communications. Compared to the conventional power system, the smart grid is designed to integrate millions of smart sensors and advanced computing technologies into the whole grid [2]; it can efficiently realize real-time automatic control, intelligent regulation, online analysis and decision making, cooperative interaction, and other advanced functions of the power grid. One feature of the smart grid is the high share of renewable generation, which poses a threat to its reliability due to the renewable intermittency. One widely adopted solution to this problem is to employ the energy storage system (ESS) in microgrids. Since wind and photovoltaics power are nondispatchable parts, the dispatching strategy of ESS becomes an important component in the smart grid with renewable generation.

Most existing energy storage planning approaches rely on historical data or highly precise generation/load forecast [3, 4]. Current power forecasting methods can be broadly categorized into two types: methods based on statistical analysis [5] and methods based on artificial intelligence (AI) algorithms [6]. For methods in the first category, the Bayesian theory, multivariable linear regression, and autoregressive moving average (ARIMA) are often employed [7, 8]. These methods are better suited for fitting data that have periodic features; the high percentage of renewable energy sources considerably increases the randomness of power variations, making methods in this category less suitable for applications in smart grids. Methods based on AI algorithms, on the other hand, are theoretically favorable in predicting an output for systems with high nonlinearity and complex dynamic properties. In particular, a recurrent neural network (RNN) [9] helps handle nonlinear problems and capture more dynamic relationships between the input and the forecasted output compared to the artificial neural network (ANN) [10], and the long short-term memory (LSTM) unit proposed in [11] further improves its performance in the prediction of time series data.

A noteworthy concern is that all the abovementioned methods require sufficient training data. The heavy reliance on information networks leads to higher cyber risks for smart grids [12]. In the event of a cyber-attack, the local database could be tampered and lost, which leads to serious consequences [13]. For instance, Ukraine's electricity supply system was hacked in 2017 through an attack on the data aggregator, which is the central node containing data from data collection base stations [14]. This event caused massive power outages, paralyzed connected nodes, and prevented the control center from accessing customer load data in time for power control and scheduling operations. It is of great interest to reduce the grid control centers' heavy reliance on local users' data, so that the day-ahead or long-term power dispatch would not be blocked by insufficient data. The primary issue that needs to be addressed is load forecasting based on inadequate local data.

Transfer learning (TL) [15, 16] is a suitable tool for addressing the challenges discussed above. It is of great value to introduce transfer learning in power systems to efficiently utilize resources from different regions, discover the commonality of different datasets, and establish transfer learning-based forecasting methods. The key idea of transfer learning is to use the existing experience to solve similar tasks, exploit similarities between data and models, and apply the trained content to new tasks. Specifically, transfer learning allows the use of knowledge in the dataset with complete labels (i.e., the source domain) to solve problems in the dataset with missing labels (i.e., the target domain), using a trained model with good generalization capability [17]. The research in [18] presents the outstanding contribution of transfer learning in the field of image processing. Innovative breakthroughs have also been made in the field of classification and target detection [19] in recent years. Lu et al. [20] proposed a general transfer learning-based framework for load forecasting with limited data. The influence of adopting different kernel functions in transfer learning for fault diagnosis is studied by Li et al. in [21]. Scholars in [22] investigate the superiority of transfer learning in extracting features and aim to predict the wind speed in different environments. Yin et al. [23] proposed a hybrid transfer learning-based wind power forecasting model. Unfortunately, the potential relationship between statistical properties in time series and transfer learning is ignored in these works.

**Figure 1:**
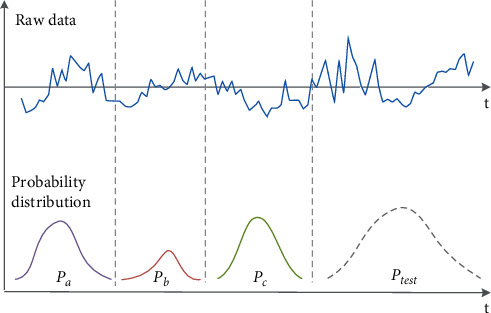
The temporal covariate shift phenomenon.

One critical issue for developing transfer learning-based forecasting methods is how to train a forecasting model with strong generalization capabilities. Many published forecasting methods for smart grids are based on the assumption that historical data follow the same distribution. The scholars in [24] made great improvements in load forecast based on machine learning in certain areas, but the generalization ability of their proposed method is not very promising since the differences between distributions of data are not considered. In a typical grid, especially those with high penetration of the renewable system that introduces high stochasticity, the distribution of the data in the temporary structure changes over time. Consider the illustrative graph in Figure 1, the probability distribution of *P*_*x*_ varies for different intervals, and the temporal covariate shift phenomenon happens after adding a new segment of data, where *P*_*a*_ ≠ *P*_*b*_ ≠ *P*_*c*_ ≠ *P*_*test*_. Here, the aforementioned issue is embodied in two aspects. First, how to build an adaptive prediction model to weaken the effect of covariate shift and accommodate the diversity of sample data. Second, how to develop a probability distribution algorithm to minimize the divergence between the distribution for different intervals.

Load prediction provides basic data for generation planning, day-ahead market offers, and intraday market trading, and it is important for the economic dispatch of power system. In the wind/photovoltaic/energy storage complementary microgrid, the generation plan of energy storage is the only dispatchable part. Researchers in [25] proposed a general method for the capacity and power of energy storage batteries and constructed a capacity allocation scheme for energy storage batteries, researchers in [26] introduced the control and communication technology, and operation principle of cloud energy storage based on the Irish power system. At present, the research on shared energy storage is in its initial stage, and the existing work takes shared energy storage systems as the main research object to analyze the business model and profitability of shared energy storage systems, while in-depth research on the charging and discharging behavior and economic benefits of users' participation in the shared energy storage system is limited. This paper introduces shared energy storage plants among different user groups and establishes an optimal scheduling model with the objective to minimize daily operation cost of user groups.

The main contributions of this paper are summarized as follows:

1. To address the challenge that the generation center fails to develop power planning for grid operation due inadequate local user data, we propose a power forecasting method based on transfer learning, where data from the source domain can provide valuable reference information. Additionally, case studies provide a detailed analysis of how to choose the appropriate source domain, and the effect of negative transfer on model performance is analyzed.
2. This work proposes to model the time series of load prediction from the covariate shift perspective. To train a forecasting model with strong generalization capability with transfer learning, we generate a combination mode where the probability distributions of time series vary for different intervals, and an optimal split method is proposed to ensure that the segments being divided are the most dissimilar ones. The temporal distribution matching algorithms are proposed to minimize the divergence between the distribution for different intervals. Dynamic programming (DP) is applied to optimize the optimal division points. The case study shows that the maximum improvement of the proposed forecasting method is up to 52.8% in mean absolute percentage error (MAPE) compared to other transfer learning-based methods, and up to 64.4% compared to the traditional method.
3. With the accurate load prediction obtained from (1) and (2), a novel shared energy storage station (ESS) concept is proposed to form a framework for optimal scheduling based on the transfer learning method. The case study shows that the proposed framework can reduce the overall operating cost of the microgrid and maximize the benefit of grid operation, thus addressing the issue of energy curtailment [27] as well as the high cost of energy storage.

This paper is organized as follows: Section 2 presents the structure of the transfer learning-based forecasting algorithm with the limited data set, and modeling time series from a covariate shift perspective, which is critical to determining the generalization ability of forecasting models.  Section 3 proposes a framework of optimal dispatch planning for the distributed microgrid, a shared energy storage station (ESS) is formed as a solution for multisource power grid scheduling, and the dispatchability of the cyber-attacked area is analyzed when using the proposed optimal economic dispatching method. The performance of transfer learning in addressing fragmented test data of the target domain is then developed. Moreover, using data obtained from the proposed forecasting method, the economic analysis of the proposed energy storage station is shown in Section 4. Finally, the main findings are included in Section 5.

## 2. Methodology of the TCS-Transfer Learning Model

### 2.1. Transfer Learning-Based Structure

In most machine learning tasks, the training and test sets come from the same feature space and are subject to the same probability distribution. When the actual conditions are not satisfied, it takes a lot of resources to collect data from the target domain to retrain a model. In particular, in a feature-rich smart grid, retraining the model becomes inefficient and time-consuming, which can seriously disrupt the schedule of the generation center, transfer learning [28] is a new solution to this problem.

Maximum mean discrepancy (MMD) is applied in this paper to define a more specific formula for the TL problem:

(1) $$\begin{array}{c} f^{*}=\underset{f\in H}{\arg\min}\frac{1}{N_{s}}\sum_{i}^{Ns}l\left(f\left(x_{i}\right),y_{i}\right)+\xi MMD\left(D_{s},D_{t}\right), \end{array}$$

where *H* represents the vector space that satisfies the objective function, *N*_*s*_ means the number of samples of the source domain *D*_*s*_, and *D*_*t*_ means the source domain and target domain. *N*_*t*_ represents the number of samples of the target domain, and *l*(*x*, *y*) denotes the loss function suitable for different structures, and *x* and *y* represent the sample and label, respectively. Argmin denotes the value of the variable that minimizes the objective function. *ξ* represents the metric coefficient. This paper selects root mean squared error (RMSE) as the measurement of error. It is considered that MMD is one of the most widely used distance metrics in TL among many statistical measures, which is an effective way to measure the correlation between any two different domains *D*_*i*_ and *D*_*j*_, and it can be formulated using the following equations:

(2) $$\begin{array}{c} MMD\left[F,D_{i},D_{j}\right]={\left[\frac{1}{m\left(m-1\right)}\sum_{i\neq j}^{m}k\left(x_{i},x_{j}\right)+\frac{1}{n\left(n-1\right)}k\left(y_{i},y_{j}\right)-\frac{2}{mn}\sum_{i,j=1}^{m,n}k\left(x_{i},y_{j}\right)\right]}^{1/2}, \end{array}$$

(3) $$\begin{array}{c} k\left(x,x^{\prime }\right)=e^{-{\left\|x-x^{\prime }\right\|}^{2}/2\sigma^{2}}, \end{array}$$

where *D*_*i*_ and *D*_*j*_ represent any two different domains, and their sample sizes are *m* and *n*. *k*(·, ·) represents the Gaussian kernel function. Radial basis kernel function *k*(*x*, *x*′) is used for mapping to the higher dimensional spaces. *x*′ is the center point of the kernel function, *σ* denoted the expectation, which controls the range of action of the Gaussian kernel function; the larger its value, the larger the local range of the influence of the Gaussian kernel function. *𝔽* denotes the unit ball in the regenerative nuclear Hilbert space (RNHS).

It is worth noting that not all source domains are suitable to be selected, Krizhevsky et al. [29] provides an in-depth analysis of the role of pretraining models for transfer tasks, a pretraining model can be used as benchmark models for the task of the target domain, researchers in [30, 31] also demonstrate that for datasets with different distributions, pretraining in an appropriate source domain can greatly improve the accuracy of the results, and source domain that has a weak correlation with the target domain may cause a negative transfer phenomenon. Therefore, it is essential to analyze the discrepancy between the *𝒟*_*s*_ and *𝒟*_*t*_; this paper measures the difference by calculating the MMD value, and the source domain can be selected for pretraining if the MMD is below the preset threshold value.

### 2.2. Problem Formulation of Temporal Covariate Shift (TCS)

The variate {*x*_*i*_} of the time series is assumed to follow the same probability distribution in most existing prediction methods, it may have achieved satisfactory results on specific datasets, such as the load of a stand-alone device in a traditional predict scene, which is relatively uncomplicated in its diversity. However, this assumption is not realizable in the actual application due to the huge amount of data and features, the variation of data distributions with the time changing cannot be ignored. Thus, our problem can be formulated as follows: split a given time series *𝕊* with *m* labeled dataset into *k* segments with the most dissimilar distribution {*𝕊*_1_ … *𝕊*_*k*_}.

(4) $$\begin{array}{c} \left\{\begin{array}{c} S=\left\{S_{1},\ldots S_{p}\ldots S_{q}\ldots ,S_{k}\right\},\\ \sum S=m. \end{array}\right) \end{array}$$

With reference to the definition of covariate drift [32] in the classification field, the temporal covariate shift can be presented such that the whole intervals in the same period *i* follow the same probability distribution *P*_*𝕊*_*p*,*q*__(*x*, *y*). The distribution will be different when the time period changes *P*_*𝕊*_*p*__(*x*) ≠ *P*_*𝕊*_*q*__(*x*) (*p* ≠ *q* ∈ (1, *k*)). To train a prediction model with excellent generalization performance under temporal covariate shift, the main issue is to capture the common knowledge shared among different periods of *𝕊*_*p*,*q*_.

**Figure 2:**
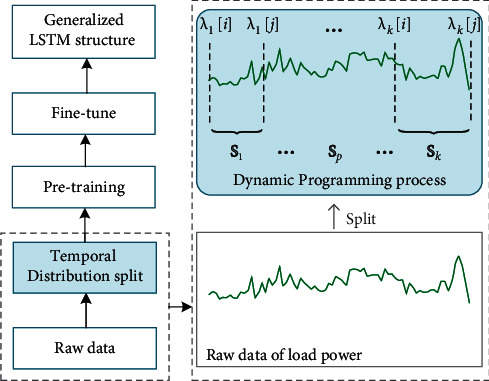
Schematics of the proposed predicting structure based on transfer learning.

According to the principle of maximum entropy [33, 34], finding intervals that are the most distinct from each other can help maximize the capacity of shared information within a time series under temporal covariate change issues. It is fair to make distributions of each interval as diverse and feasible to maximize the entropy of the overall distributions of an array. This enables for more generic and adaptable future data modeling. Worst-case training using the original sequence enables the model to cope with the stochastic nature of the unknown data.  Figure 2 shows the structure of the proposed predicting method based on transfer learning, and the primary task is to split the time series into *k* segments. The splitting problem of the time series in (5) is solved by the greedy algorithm.

(5) $$\begin{array}{c} \underset{m_{1},\ldots m_{k}}{\max}\frac{1}{k}\sum_{1\leq \lambda_{i}\neq \lambda_{j}\leq k}MMD\left(S_{p},S_{q}\right),\\ s.t.\forall i,\;\Delta_{1}\leq \left|\lambda \left[j\right]-\lambda \left[i\right]\right|\leq \Delta_{2}, \end{array}$$

where array *λ*[1...*m*] represents a set of time series, *m* represents the length of it, *MMD*(·, ·) means the distribution-based distance function to measure the distance between distributions of any two segments *𝕊*_*p*_, *𝕊*_*q*_, *λ*[*c*], *c* ∈ [*i*, *j*] denotes the coordinate position of the cut at each time. *λ*[*i*] and *λ*[*j*] are the left and right endpoints of the segment to be segmented, respectively. *λ*[*j*]_−1_ is the right endpoint of the previous interval and *λ*[*i*] is the start point of the next interval. Δ_1_ and Δ_2_ represent predefined constants to prevent trivial solutions.

### 2.3. Matching Process and Fine-Tune

**Figure 3:**
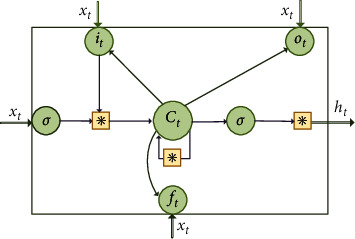
LSTM unit structure.

The proposed method is designed to acquire the common information shared by distinct intervals by comparing their probability distributions, this section presents the process of how to pretrain a model after obtaining the optimal split intervals in raw data. In comparison to approaches that simply depend on local or statistical knowledge, the pretraining model ℳ can produce a nice generalization on unknown datasets of the target domain. The pretraining issue formulated in Section 2.2 can be solved by the domain generalization (DG) [35] method, and the distribution matching loss of the network can be established as follows:

(6) $$\begin{array}{c} \theta^{*}=\text{arg}\underset{\theta }{\min}\sum_{i=1}^{k}{ℒ}_{\text{pre}}\left(y_{i},\hat{y_{i}}\right)+\xi \sum_{1<i<j<k}MMD\left(S_{i},S_{j}\right), \end{array}$$

where ℒ(·, ·) denotes the MSE loss in the source domain, and *𝕊*_*i*_ and *𝕊*_*j*_ are any two different intervals of *λ*[1 … *m*]. In the proposed method, LSTM is employed as the main body of the network structure. Due to the special memory unit of LSTM, potential relationships between data in a time series can be preserved, which can provide high accuracy for the prediction results. Compared to the conventional recurrent neural network algorithm, which contains only one state *h*_*t*_, the LSTM structure introduces cell states *c*_*t*_ to develop potential relationships in a long time series. The LSTM structure can be formulated as follows:

(7) $$\begin{array}{c} i_{t}=\sigma \left(W_{i}*\left[h_{t-1},x_{t}\right]+b\right),\\ {\tilde{c}}_{t}=\tanh\left(W_{c}*\left[h_{t-1},x_{t}\right]+b\right),\\ c_{t}=f_{t}⊙c_{t-1}+i_{t}*{\tilde{c}}_{t},\\ o_{t}=\sigma \left(W_{o}\left[h_{t-1},x_{t}\right]+b\right),\\ h_{t}=o_{t}*\tanh\left(c_{t}\right), \end{array}$$

where *x*_*t*_ is the input data, *W*_*i*,*c*,*o*_ represent the weight matrices, *i*_*t*_ , *f*_*t*_, and *o*_*t*_ are the input, forget, and output gates of the LSTM structure, respectively. *b* represents the bias value. *c*_*t*−1_ denotes the state of the memory cell. The candidate value ${\tilde{c}}_{t}$ is generated by tanh layer. *h*_*t*_ presents the output value, *h*_*t*−1_ represents the output value of the previous unit, the sigmoid function is denoted as *σ*, and *∗* represents the dot product. ⊙ is the element-wise product.  Figure 3 shows the structure of LSTM; the type of data being inputted is described in Section 4.

**Figure 4:**
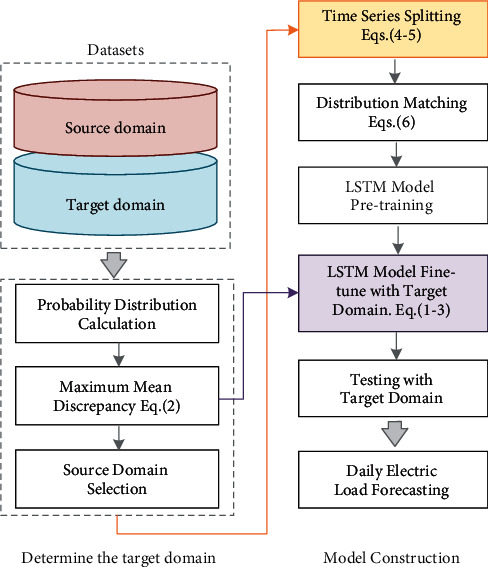
Power forecasting structure based on the TCS-transfer learning model.

Figure 4 shows the flowchart of the proposed transfer learning-based forecasting structure. The implementation steps of the whole network are as follows:

1. Start by collecting the raw data of power fluctuations from neighboring cities and calculating the probability distribution of the candidate datasets
2. Calculate the distance between the source and target domains according to eq. (2) and select the appropriate source domain as the input data of the pretrain model
3. For the selected source domain, it is first divided into *k* most dissimilar segments using the proposed dynamic programming-based method according to eqs. (4) and (5).
4. The *k* segments are considered as different domains. Eq. (6) is used as the new loss function in the model with LSTM as the main network. After obtaining a prediction network with strong generalization capability, the target domain with very less data is used to fine-tune by using Eq. (1)–(3).

The proposed temporal covariate shift issue focuses on an easily neglected problem in time series Dynamic programming solves the optimal spilt problem of the time series, considering the fragment as *k* independently distributed individuals. The generalization ability of the pretraining model using source domain data is greatly improved by taking the differences between these segments as an additional consideration in the loss function. The deep LSTM network can be trained with less learning time while overcoming the problem of lacking sufficient local data as training datasets.

## 3. Optimal Dispatch Planning for Distributed Microgrid

### 3.1. Smart Grid with Multiple Sources of Energy Supplies

**Figure 5:**
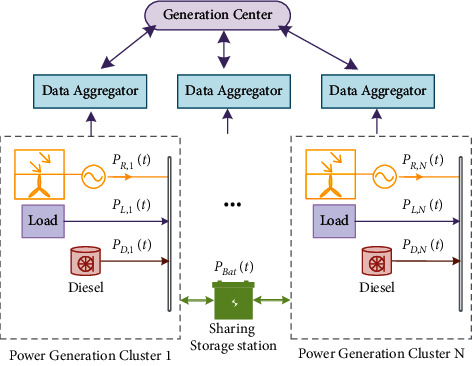
Conceptual framework of the multienergy complementary power generation system.

Smart grids are increasingly used in recent years for both residential and industrial purposes. In many microgrids, the hybrid wind-solar generation plant is employed owing to the complementary nature of wind and solar generation patterns. Therefore, the smart grid with renewable generations is studied in this paper as shown in Figure 5. From the perspective of the Internet of Things (IoT) network structure of the smart grid, the data is collected by a large number of smart meters in the users' network, encrypted by the gateway, gathered by the data aggregator, and then transmitted to the control center, and this network data distribution characteristic is called the “funnel effect” [36]. In a smart grid with deeply integrated information systems, the form of fault propagation has more possibilities. When a node in the network fails, it will trigger the failure of the related nodes, and the existing scheme requires all users to work collaboratively. As long as one user fails, the whole system cannot operate normally. The transfer learning-based forecasting method proposed in this paper can substantially reduce the dependence of generation centers on the adequacy of local data.

The generation plant of the microgrid is divided into *N* clusters, each having wind turbine and solar photovoltaic (PV) systems. The output power of the wind/PV system in the *j*-th cluster can be expressed as follows:

(8) $$\begin{array}{c} P_{G,j}\left(t\right)=P_{W.j}\left(t\right)+P_{PV,j}\left(t\right)+P_{D,j}\left(t\right),\;j\in \left\{1,2,\ldots ,N\right\}, \end{array}$$

where *P*_w,*j*_, *P*_*PV,j*_, and *P*_*D,j*_ represent the output power of wind turbines, photovoltaic systems, and diesel systems, respectively. In modern microgrids, generators are usually used as backup power sources, the term *P*_*D*,*j*_(*t*) in (8) can be ignored if no power failure occurs. It can be further expressed in per unit (p.u.) value denoted by *P*_*∗G*,*j*_(*t*) as eq. (9), where *P*_*G*,base_(*t*) denotes the base power in the DC bus, *P*_*G*,*j*_(*t*) is the actual power value of the DC bus, and*P*_Bat_(*t*) represents the power of the battery.

(9) $$\begin{array}{c} \left\{\begin{array}{c} P_{*G,j}\left(t\right)=\frac{P_{G,j}\left(t\right)}{P_{G,\text{base}}\left(t\right)},\\ P_{PV,j}\left(t\right)+P_{W.j}\left(t\right)+P_{D,j}\left(t\right)+P_{\text{Bat}}\left(t\right)=P_{L,j}\left(t\right). \end{array}\right) \end{array}$$

### 3.2. Operation Mode and Market Rules of a Shared Station

The concept of a shared energy storage plant is shown in Figure 5, in which the operator of an energy storage station uses the financial advantage to establish a large shared energy storage plant among a group of customers, and unifies the operation and management of the energy storage station to provide shared energy storage services to multiple customers in the same distribution network area. The generation center forecasts the load based on historical electricity consumption data and plans to use shared energy storage plants for charging and discharging, which minimizes the economic cost of operation of the storage devices and saves the customer the investment cost of installing and maintaining the storage devices. Power market rules require the dispatch plan submitted to the grid operator 24 hours ahead.

**Figure 6:**
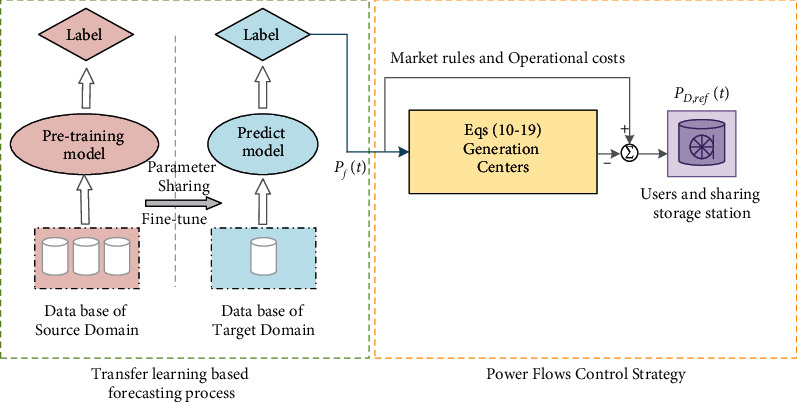
Schematics of the proposed optimal power planning procedure.

Figure 6 depicts the framework of the proposed power dispatch schedule. The main purpose of this work is to solve the issue that the predicting process cannot operate due to insufficient local data, thus, the power data of the neighbor area is employed to train highly generalizable models, and the precise prediction model can be obtained after fine-tuning with few local data.

The generation center of the energy shared storage power station transmits the remaining power of users who need charging directly to users who need discharging according to the charging demand and discharging demand of each user at each time. The generation station of energy storage station can utilize the complementary nature of customers' power consumption behavior, i.e., the difference in power consumption behavior of the same customers at different times and different customers at the same times, and can maximize the investment in the least amount of energy storage to meet customers' demand for energy storage use. The specific optimization strategy of shared storage stations is detailed in Section 3.3.

### 3.3. Optimal Economic Scheduling of Shared Energy Storage Station

**Figure 7:**
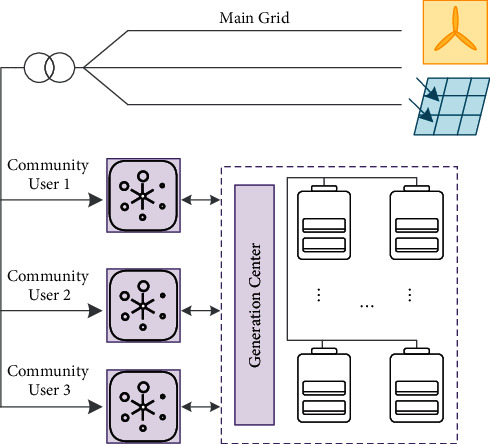
Schematic diagram of the shared energy storage station.

Having explained the forecasting method and the possible operational modes of the microgrid, the next task is to develop the dispatch strategy for the shared ESS to economically benefit users. The high investment cost of energy storage is the main reason that limits the application of energy storage technology on the demand side of the grid. This paper proposed the concept of a shared energy storage station, as shown in Figure 7, which is applied to the economic optimization scheduling of regional users, and the minimum daily operating cost of the user group is achieved by coordinating the charging and discharging power of the users. The energy storage system allows users to store electricity during the grid valley hours and release it during peak hours, thereby decreasing electricity costs and relieving the pressure on regulating the peak load. According to the charging demand and discharging demand of each user in each period, the generation center will deliver the remaining electric energy of the user who needs to discharge directly to the user who needs to charge. If the total charging and discharging demand of users in the same time period is discharged, the regulation center will decide whether the users' electricity needs to be purchased by the main grid or stored in the shared power station according to the electrovalence at that time.

Based on the predicting method proposed in the previous sections, this part develops a scheme to obtain the minimum grid operating cost through proper charging and discharging of the shared energy storage station. By using the shared energy storage station, the user saves the investment costs for the installation and maintenance of energy storage devices. Users pay the service fee to the generation center in exchange for shared energy storage services. The service fee means the users pay to the generation center when they use the shared energy storage stations for charging and discharging, it is set as 0.16$/kWh.

#### 3.3.1. Objective Function of the Optimization Model

According to the market rules presented in Section 3.2, the user group connected to the shared energy storage station uses the typical daily operating cost optimization as the objective function to determine the capacity, the maximum charging and discharging power of the energy storage station, and the charging and discharging power of the storage station for each time period of the user. The daily operating cost of the customer group includes the cost of electricity purchased from the grid and the service fee paid to the energy storage station.

(10) $$\begin{array}{c} \min C=C_{g}+C_{s}, \end{array}$$

where *C* represents the daily cost of electricity for the user community; *C*_*g*_ and *C*_*s*_ denote the cost of electricity purchased by the customer from the grid and the service fee paid to the energy storage station, respectively.

(11) $$\begin{array}{c} \left\{\begin{array}{c} C_{g}=\sum_{i=1}^{N}\sum_{t=1}^{T}\rho \left(t\right)\cdot P_{G,i}\left(t\right)\cdot \Delta t,\\ C_{s}=\sum_{i=1}^{N}\sum_{t=1}^{T}\delta \left(t\right)\cdot \left[P_{E,D,i}\left(t\right)+P_{E,C.i}\left(t\right)\right]\cdot \Delta t, \end{array}\right) \end{array}$$

where *N* represents the serial number of the user group connected to the same shared energy storage station, user groups of three areas are selected as case studies in this paper (*i* ∈ [1,3]); *ρ*(*t*) ($/*kW* · *h*) represents the price that the users purchase electricity from the grid. *T* denotes the scheduled time periods; *P*_*G*,*i*_(*t*) indicates the power value purchased by the user *i* from the grid at a given time interval *t*; Δ*t* represents the unit time length of the power scheduled; *δ*(*t*) is the service fee of the shared energy storage station; *P*_*E*,*D*,*i*_(*t*) and *P*_*E*,*C*,*i*_(*t*) are discharge power and charging power of energy storage station at time *t*, respectively.

#### 3.3.2. Constraint Condition

The constrains should be met by the proposed power planning model. They include the following: electrical power balance constraints [26] and operational constraints of the energy storage stations:

(1) Power balance constrain of the whole grid:
  (12) $$\begin{array}{c} P_{G}\left(t\right)+P_{PV,i}\left(t\right)+P_{W,i}\left(t\right)+P_{ESS}\left(t\right)-P_{L,i}\left(t\right)=0,\\ P_{ESS}=P_{E,D,i}\left(t\right)-P_{E,C,i}\left(t\right)=\text{MAX}\left(P_{W,i}\left(t\right)+P_{PV,i}\left(t\right)-P_{L,i}\left(t\right)\right), \end{array}$$
  where *P*_*G*_(*t*) is the power purchased from the main network; *P*_*PV*,*i*_(*t*) represents the output power of the PV system of the *i-th* user in time interval *t*; *P*_*W*,*i*_(*t*) is the wind power of the *i-th* user in time interval *t.P*_*L*,*i*_(*t*) denotes the load power of the *i-th* user, which is a predicted value obtained by the method proposed in the previous section; *P*_*ESS*_ is the power of the energy storage station, which satisfies the maximum power difference between generation and load in the grid for any period of time; *P*_*E*,*D*,*i*_(*t*) and *P*_*E*,*C*,*i*_(*t*) represent the power under discharging and the charging status of the energy storage system.
(2) Charging and discharging power constraints for a shared energy storage station:
  (13) $$\begin{array}{c} \left\{\begin{array}{c} 0\leq P_{E,D,i}\leq P_{\max}\delta_{ESS\_D}^{\max},\\ 0\leq P_{E,C,i}\leq P_{\max}\delta_{ESS\_C}^{\max},\\ \delta_{ESS\_D}^{\max}=\frac{SOC-SOC_{\min}}{SOC_{l}-SOC_{\min}},\\ \delta_{ESS\_C}^{\max}=\frac{SOC_{\max}-SOC}{SOC_{\max}-SOC_{h}}, \end{array}\right) \end{array}$$
  (14) $$\begin{array}{c} \left\{\begin{array}{c} SOC=\left(SOC_{0}-\frac{1}{Q\int_{0}^{t}\eta Idt}\right)\times 100\%,\\ SOC_{\min}\leq SOC\leq SOC_{\max}, \end{array}\right) \end{array}$$
  where *P*_max_ is the rated maximum power of the charging and discharging power of the energy storage station; *δ*_*ESS*_*D*_^max^ and *δ*_*ESS*_*C*_^max^ are defined as the discharging and charging state factor, respectively, which ensure that the energy storage is not in an overcharged and discharged state; *SOC*_min_ and *SOC*_max_ represent the operating range of the energy storage; *SOC*_*l*_ and *SOC*_*h*_ represent the optimal working interval for energy storage; *SOC* denotes the state of the charge value of the energy storage; *SOC*_0_ is the initial state of charge; *η* is the charge and discharge efficiency; *Q* represents the electric charge quantity; *I* is the battery current.
(3) Power balance constrain of the energy storage station:
  (15) $$\begin{array}{c} \sum_{i=1}^{N}\left|P_{E,D.i}\left(t\right)-P_{E,C.i}\left(t\right)\right|=\left|P_{D}\left(t\right)-P_{C}\left(t\right)\right|, \end{array}$$
  (16) $$\begin{array}{c} \left\{\begin{array}{c} \delta_{ESS\_D}^{\max}+\delta_{ESS\_C}^{\max}\leq 1,\\ \delta_{ESS\_D}^{\max}\in \left\{0,1\right\},\delta_{ESS\_C}^{\max}\in \left\{0,1\right\}. \end{array}\right) \end{array}$$
The charging and discharging power of each user in a time period *t* needs to be balanced with the charging *P*_*C*_(*t*) and discharging *P*_*D*_(*t*) power of the energy storage station.

#### 3.3.3. Resolve Method

The few nonlinear terms in the abovementioned constraints can significantly increase the difficulty and time during the solving process of numerically solving the aforementioned optimization problem. To overcome this challenge, Big-M [27] is adopted to linearize the nonlinear constraints in this work. The user scheduling model based on shared energy storage stations can be converted to a mixed integer linear programming problem. Thus, (15) can be reformulated as follows:

(17) $$\begin{array}{c} \left\{\begin{array}{c} 0\leq P_{E,D,i}\leq P_{\max},\\ 0\leq P_{E,D,i}\leq \delta_{ESS\_D}^{\max}M,\\ 0\leq P_{E,C,i}\leq P_{\max},\\ 0\leq P_{E,C,i}\leq \delta_{ESS\_C}^{\max}M,\\ \delta_{ESS\_D}^{\max}+\delta_{ESS\_C}^{\max}\leq 1,\\ \delta_{ESS\_D}^{\max}\in \left\{0,1\right\}\delta_{ESS\_C}^{\max}\in \left\{0,1\right\}, \end{array}\right) \end{array}$$

where the value of *M* is determined as 10^8^ in this paper.

## 4. Case Study on the Smart Grid System

### 4.1. Setup and Experimental Result of the Proposed Forecasting Method

**Figure 8:**
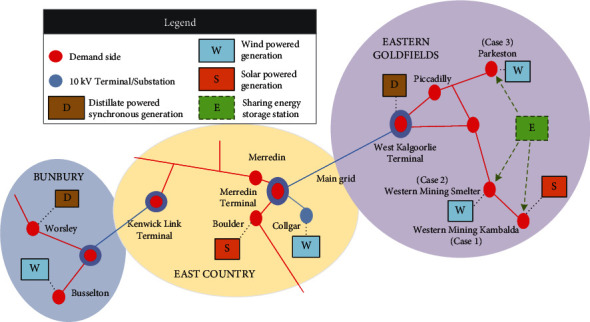
Geographical locations of the case study.

Field load data of three regions from Western Australia are utilized for the case studies, covering the time range of May 1, 2015, to July 1, 2021. A map of the regions of the presented case studies is shown in Figure 8, where Case 1 and Case 2 are two typical industrial type electricity consumption areas, and Case 3 is the residential user group. Each node represents a region of independent microgrids that do not interfere with each other and are powered primarily by renewable energy. It is noted that 80% of the historical data are used as the training set and the remaining 20% are used as a validation set. The train/valid sets are structured with a ratio of 8 : 2, the test set is from the real load data from users. When load forecasting is implemented, 168 steps are batched together to train the model and predict the net power in the next 24 steps, and the sampling interval is 1 hour and the forecasting horizon covers 24 hours. The input feature mainly consists of the population, temperature, and calendar data which contain the season, number of holidays, and weekdays. The features are summarized in Table 1.

**Figure 9:**
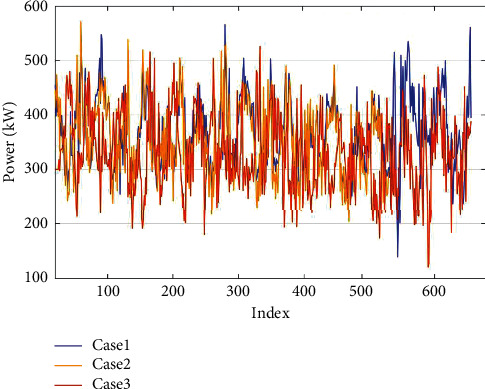
Raw data of three cases.

Figure 9 shows the raw data collected from three cases presented in Figure 8. As shown by the yellow line, part of the data in the western mining smelter (Case 2) is absent because data loss occurred due to aggregator overload. The common deep learning-based predicting models are not universal for different data sources, and each region needs to train a locally applicable predicting model based on its own database. According to the proposed transfer learning-based model, after comparing the MMD value of each database associated with the neighboring grid, the database of a microgrid in western mining Kambalda is chosen as the source domain.

To demonstrate the performance of the proposed structure, this work compares the proposed TCS-transfer learning model with four categories of methods listed as follows:

- Traditional time series forecasting model LSTM with classical gradient descent: there are also BP [37], ANN, and CNN [38] belonging to this category, and the most applicable time series predicting model–LSTM is selected in this category
- The latest time series structure ELM [39] without backward propagation: the convergence time is substantially reduced and the training efficiency has been greatly improved. The first two categories are classical methods of time series forecasting.
- Variants of popular domain adaptation methods include MEDA-LSTM [40] and MMD-RNN [41], which are also based on the concept of transfer learning
- A branch of transfer learning is that a transformer with an attention mechanism [42, 43] has a stronger generalization capability than the classical method. This paper uses 6 encoder blocks of a transformer and 8 heads for self-attention.

The relevant parameters are listed in Table 2. The parameters that produce the best performance for each model are tuned by K-fold cross-validation. The following comparison discusses the effectiveness of the proposed TCS-transfer learning model mainly from two aspects: first, the power grid of the western mining smelter (Case 2) is with missing data, thus the traditional methods such as LSTM and ELM included in categories i and ii failed to forecast the load power due to the fragmented dataset in Case 2. For cases of missing data in the test set, transfer learning is the only method that can solve the problem. The first part of the comparison in this section uses different transfer learning based approaches to predict the load power of Case 2. Second, this paper presents that the proposed method is also suitable for the time series forecast with a complete dataset. The comparison group of Case 1 and Case 3 contains both traditional and transfer learning based methods.

### 4.2. Performance of Transfer Learning in Addressing Fragmented Test Data in the Target Domain

**Figure 10:**
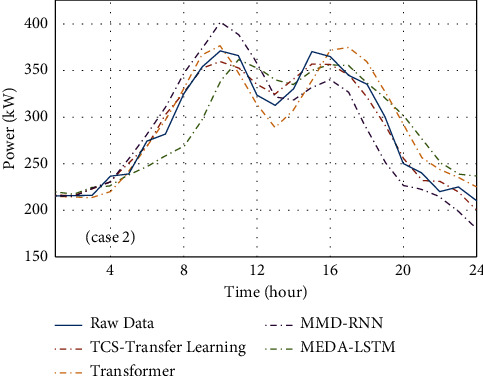
Accuracy comparison between TCS-transfer learning and other methods based on the database of Case 2.

This section mainly discusses the differences between the proposed TCS-transfer learning model and other transfer learning-based methods in dealing with load forecasting problems. In the first set of experiments in this paper, transformer, MMD-RNN, and MEDA-LSTM are used to compare the superiority of the proposed approach among transfer learning-based learning methods, the forecast load result is shown in Figure 10.  Table 3 presents the prediction error of each method with RMSE and MAPE. These prediction errors are evaluated by

(18) $$\begin{array}{c} RMSE=\sqrt{\frac{\sum_{t=0}^{n}{\left({\hat{x}}_{t}-x_{t}\right)}^{2}}{n}}, \end{array}$$

(19) $$\begin{array}{c} MAPE=\frac{1}{n}\sum_{t=0}^{n}\frac{\left|{\hat{x}}_{t}-x_{t}\right|}{x_{t}}\times 100\%, \end{array}$$

where ${\hat{x}}_{t}$ denotes the predicted value of samples, *x*_*t*_ is the actual value of samples, and *n* represents the number of samples.

The convergence times of different forecasting methods are listed in Table 4; it takes 0.34 s for convergence with regard to the proposed TCS-transfer learning structure, which is a 60.4% increase compared with the MMD-RNN. The prediction accuracy (MAPE) is improved by 52.8% over the RNN-based method, considering GPU cycles with Intel Core i9-12900K. Although the proposed method is slightly slower than the transformer in terms of convergence speed, it has a great improvement in prediction accuracy.

**Figure 11:**
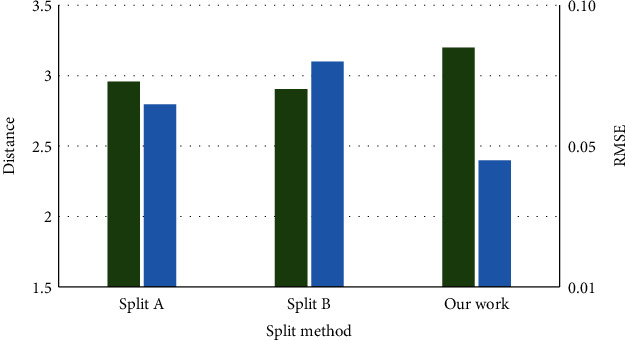
Different split methods.

To evaluate the effect of different splitting methods on the training results, two additional methods of dividing the time series in Figure 11, i.e., splits A and B, were designed in this work. Split A denotes that the sequence is randomly divided into *k* segments; split B is the method where all intervals are with similar distributions, which is the opposite of our proposed split method. The proposed split method has the objective of minimizing the cost function in (5). The “distance” on the *Y*-axis means the distribution distance MMD with the green line and the RMSE denote the error with the blue line. As a result, it is critical that we divide the periods according to the worst case, where the distributions are the most varied.

### 4.3. Results from the Case Study on Power Dispatch Strategy

**Figure 12:**
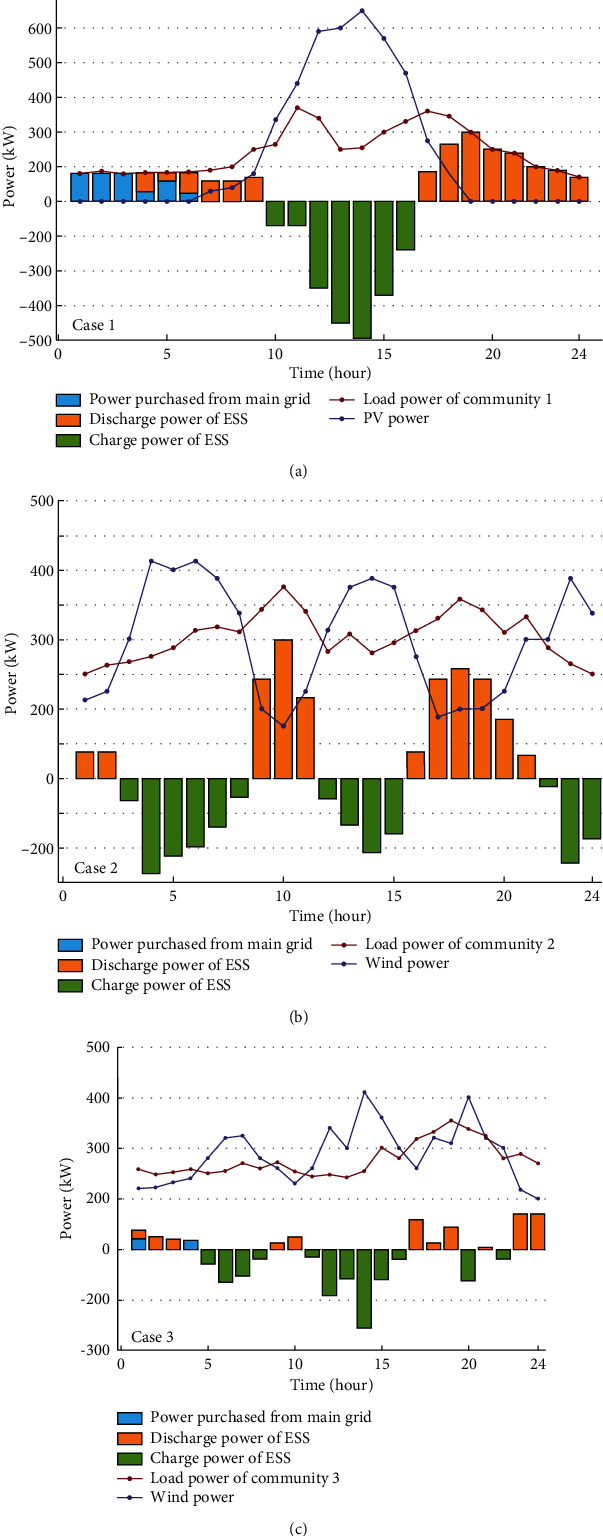
Power load balance curves of communities 1–3.

Figure 12 shows the results of power dispatch solved according to eqs. (10)–(19), whose location are illustrated in Figure 8. It can be observed from Case 1 in Figure 12 that the PV output power of the user group in the morning is less than the demand side power; the demanded electricity for this period is purchased from the grid as well as using part of the shared power station considering the optimal economics. When the PV power is higher than the demanded power, the surplus power within the community is stored by the storage power plant to avoid energy curtailment. At the period of 15:00–20:00, the demand of the community cannot be met by the PV system. While the electricity pricing is high in this time period, the undersupplied energy is provided by the shared energy storage station.  Table 5 provides the electricity rate obtained from the energy provider.

**Figure 13:**
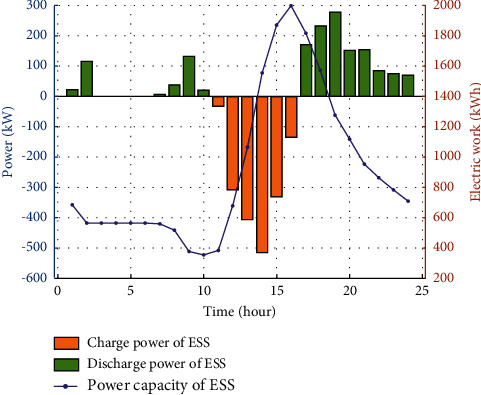
Output power and SOC curves of the shared energy storage station.

The configuration of the shared energy storage plant results in a capacity of 2,508 kWh and a maximum charge/discharge power of 637 kW. It can be observed from Figure 13 that at the period of 01:00–06:00, the electricity of users is purchased from the main grid and the power station does not provide electricity to the customer. During the 10:00–17:00, the energy storage station is in a charging state and the power rises from 382 kWh to the maximum value of 2,000 kWh, where the charging and discharging power value of the energy storage plant is negative, which means that the energy storage plant is charging; if the charge/discharge power value is positive, it denotes that the energy storage plant is discharging.

As can be seen from Figures 12-13, the electric loads of communities 1–3 have reached a balanced state, and there is no phenomenon of energy curtailment, meanwhile, the energy storage station returns to the initial operation state after one cycle of operation, ensuring the normal operation of the next cycle of the energy storage station.

Table 6 compares the daily cost of each case considering different configuration methods of energy storage, the first method presents the independent energy storage system within each user. The total operating cost is AUD 2618.6, which is 36% more expensive than our proposed planning method, and the capacity of the energy storage required by the customer is reduced by 37.3% due to the complementary nature of the customer's power consumption behavior. Moreover, the energy curtailment is well addressed and the output power of renewables is fully utilized. One limitation with shared energy storage stations is that they are prone to cause harmonics after they are connected to the grid, which can compromise power quality; therefore, more management needs to be put into safe operation when using energy storage stations.

## 5. Conclusion

This paper proposed a framework for smart grid scheduling that is less reliant on local data while capable of delivering schedules with low operating costs. Specifically, the proposed framework contains the following: (1) a power forecasting model based on deep transfer learning which can provide high-quality load prediction with limited training data; (2) a novel adaptive time series prediction method based on a neighboring area dataset that aims to train the forecasting model with strong generalization capability; (3) a day-ahead optimal economic power scheduling model considering the shared energy storage station. Results based on a case study with field load data in Western Australia showed that the maximum improvement of the proposed forecasting method is up to 52.8% in MAPE compared to other transfer learning-based methods, and up to 64.4% compared to the traditional method. The total operating cost after optimization according to the proposed method was reduced by 36.1%. These numbers indicate the proposed framework is a promising approach to solving power planning problems with incomplete datasets, in particular in addressing the cyber threats.

In the future, we plan to explore a deeper extension of TCS-transfer learning to a transformer for better performance. Moreover, this work only designed a centralized energy storage system. If multiple energy storage is needed, optimal coordination between these dispatch-oriented energy storage systems would be considered a promising area for future investigation.

## Figures and Tables

**Table 1:** Input feature of the forecasting model.

No.	Input feature	Description
**1**	No. of days	Integer
**2**	No. of holidays	Integer
**3**	No. of weekdays	Integer
**4**	Holiday length	Integer
**5**	Season	Binary
**6**	Population	Integer
**7**	Temperature	Integer

**Table 2:** Parameter set of different forecasting methods.

Model	Meaning	Value
TCS-transfer learning	Hidden layers	2
	Number of input layer nodes	4
	Number of hidden layer nodes	32
	Activation function	Sigmoid, tanh
	Learning rate	0.01
	Optimization function	ReLU
	Epochs of training	400
	Distance function	MMD
	Data preprocessing methods	Optimal splitting
	Hidden layers	2

MMD-RNN	Number of input layer nodes	4
	Number of hidden layer nodes	32
	Optimization function	ReLU
	Learning rate	0.01
	Epochs of training	500
	Distance function	MMD
	Data preprocessing methods	None
	Hidden layers	2
	Number of input layer nodes	4
	Number of hidden layer nodes	32

LSTM	Activation function	Sigmoid, tanh
	Optimization function	ReLU
	Learning rate	0.01
	Epochs of training	500

**Table 3:** Comparison between MAPE and RMSE values obtained using different forecasting methods.

Methods	MAPE (%)	RMSE
Ref. [41] MMD-RNN	6.09	0.457
Ref. [40] MEDA-LSTM	4.94	0.395
Ref. [42] transformer	4.13	0.196
The proposed TCS-transfer learning	2.87	0.042

**Table 4:** Convergence time (s) of different forecasting methods.

Methods	Time (s)
Ref. [41] MMD-RNN	0.86
Ref. [40] MEDA-LSTM	0.68
The proposed TCS-transfer learning	0.36
Ref. [42] transformer	0.21

**Table 5:** Electricity rates.

Level	Time frame	Electricity rates of the main grid [44] $AUD/kWh
Peak	Commercial users 09:00–12:00; 14:00–19:00	0.253
	Residential users 07:00–09:00; 17:00–20:00	0.386

Average	Commercial users 12:00–14:00; 19:00–24:00	0.172
	Residential users 09:00–17:00; 20:00–24:00	0.322

Trough	Commercial users 24:00–09:00	0.073
	Residential users 24:00–07:00	0.086

**Table 6:** User costs with using different energy storage configurations per day.

Optimization planning results of schemes 1 [45]				Optimization planning results of our work		
No. of user communities	Capacity of ESS (kW·h)	Maximum charge/discharge power (kW)	Operating costs ($AUD)	Power purchase from the grid (kW·h)	Service fee of shared ESS ($AUD)	Operating costs ($AUD)

Community 1	2820	621	1416.5	583	702.5	759.2
Community 2	1005	163	566.3	16	593.4	595.4
Community 3	376	78	635.8	78	311.4	318.1
Total	4001	862	2618.6	677	1607	1672.7

Optimization planning results of schemes 1: independent configuration of the energy storage system within each user. Optimization planning results of our work: each user has access to a shared energy storage station.

## Data Availability

The features data input into the predicting model used to support the findings of this study have been deposited in the following repository: (1) Exemplary Energy Partners Company. (http://www.exemplary.com.au/), (2) Office Holidays (https://www.officeholidays.com/countries/australia/2021), and (3) Australia Net Migration Rate 1950-2022 (https://www.macrotrends.net/countries/AUS/australia/net-migration). The net power data used to support the findings of this study are currently under embargo, while the research findings are commercialized. Requests for data, 6/12 months after publication of this article, will be considered by the corresponding author

## References

[1] Irena (2011). Planning for the renewable future. https://www.irena.org/energytransition.

[2] Dabbaghjamanesh M., Kavousi-Fard A., Dong Z. Y. (2020). A novel distributed cloud-fog based framework for energy management of networked microgrids. *IEEE Transactions on Power Systems*.

[3] Min Z., Wang B., Guo S., Watada J. (2021). Multi-objective prediction intervals for wind power forecast based on deep neural networks. *Information Sciences*.

[4] Victor N., Lopez D. (2020). Sl-LSTM: a Bi-directional LSTM with stochastic gradient descent optimization for sequence labeling tasks in big data. *International Journal of Grid and High Performance Computing*.

[5] Kivi M. S., Blankely B., Masters M., Bernacchi C. J. (2022). Development of a data-assimilation system to forecast agricultural systems: a case study of constraining soil water and soil nitrogen dynamics in the APSIM model. *Science of the Total Environment*.

[6] LeCun Y., Bengio Y., Hinton G. (2015). Deep learning. *Nature*.

[7] Alqahtani M. A. (2022). Cybersecurity awareness based on software and E-mail security with statistical analysis. *Computational Intelligence and Neuroscience*.

[8] Cai M., Pipattanasomporn M., Rahman S. (2019). Day-ahead building-level load forecasts using deep learning vs. traditional time-series techniques. *Applied Energy*.

[9] Cui M. S. (2022). District heating load prediction algorithm based on bidirectional long short-term memory network model. *Energy*.

[10] Luo X., Zhu X. (2021). Deep learning based forecasting of photovoltaic power generation by incorporating domain knowledge. *Energy*.

[11] Dong H. H., Gao Y., Fang Y., Liu M. (2021). The short-term load forecasting for special days based on bagged regression trees in qingdao, China. *Computational Intelligence and Neuroscience*.

[12] Sultana A., Bardalai A., Sarma K. K. (2022). Salp swarm-artificial neural network based cyber-attack detection in smart grid. *Neural Processing Letters*.

[13] Wikipedia (2015). Ukraine power grid hack. https://en.wikipedia.org/wiki/Ukraine_power_grid_hack.

[14] Lallie H. S., Debattista K., Bal J. (2020). A review of attack graph and attack tree visual syntax in cyber security. *Computer Science Review*.

[15] Pan S. J., Yang Q. (2009). A survey on transfer learning. *IEEE Transactions on Knowledge and Data Engineering*.

[16] Cui W., Zheng G., Shen Z., Jiang S. Transfer learning for sequences via learning to collocate.

[17] Bisheh M. N., Wang X., Chang S. I., Lei S. (2022). Image-based characterization of laser scribing quality using transfer learning. *Journal of Intelligent Manufacturing*.

[18] Liu Y., Liu S., Xu J., Kong X., Xie L., Chen K. (2021). Forest pest identification based on a new dataset and convolutional neural network model with enhancement strategy. *Computers and Electronics in Agriculture*.

[19] Lines J., Bagnall A. J. (2014). Time series classification with ensembles of elastic distance measures. *Data Mining and Knowledge Discovery*.

[20] Lu Y. K., Tian Z., Zhou R., Liu W. (2021). A general transfer learning-based framework for thermal load prediction in regional energy system. *Energy*.

[21] Li J. K., Lin M., Li Y., Wang X. (2022). Transfer learning network for nuclear power plant fault diagnosis with unlabeled data under varying operating conditions. *Energy*.

[22] Liang T., Zhao Q., Lv Q., Sun H. (2021). A novel wind speed prediction strategy based on Bi-LSTM, MOOFADA and transfer learning for centralized control centers. *Energy*.

[23] Yin H., Ou Z., Fu J., Cai Y., Chen S., Meng A. (2021). A novel transfer learning approach for wind power prediction based on a serio-parallel deep learning architecture. *Energy*.

[24] Li C., Liu X., Cao Y. (2015). A time-scale Adaptive dispatch method for renewable energy power supply systems on islands. *IEEE Transactions on Smart Grid*.

[25] Jiang C., Xia Z. (2022). Application of a hybrid model of big data and BP network on fault diagnosis strategy for microgrid. *Computational Intelligence and Neuroscience*.

[26] Li Y., Vilathgamuwa M., Choi S. S. (2020). Design of minimum cost degradation-conscious lithium-ionbattery energy storage system to achieve renewable power dispatchability. *Applied Energy*.

[27] Pali B. S., Vadhera S. (2020). An innovative continuous power generation system comprising of wind energy along with pumped-hydro storage and open well. *IEEE Transactions on Sustainable Energy*.

[28] Ismail Fawaz H. Transfer Learning for Time Series Classification.

[29] Krizhevsky A., Sutskever I., Hinton G. E. (2012). Imagenet classification with deep convolutional neural networks. *Proceedings of the Advances in Neural Information Processing Systems*.

[30] Long M., Wang J., Jordan M. I. (2018). Deep transfer learning with joint adaptation networks. https://arxiv.org/abs/1605.06636.

[31] Huang X., Belongie S. Arbitrary style transfer in real-time with adaptive instance normalization.

[32] Du Y., Wang J., Feng W., Pan S., Qin T. AdaRNN: adaptive learning and forecasting for time series.

[33] Tzeng E., Hoffman J., Zhang N., Saenko K. (2018). Deep domain confusion: maximizing for domain invariance. https://arxiv.org/abs/1412.3474.

[34] Wang F. Y., Zhang J., Wei Q., Zheng X., Li L. (2017). PDP: parallel dynamic programming. *IEEE-caa Journal of Automatica Sinica*.

[35] Graves A., Schmidhuber J. (2005). Framewise phoneme classification with bidirectional LSTM and other neural network architectures. *Neural Networks*.

[36] Liu W. P., Peng T., Tang R., Umeda Y. (2020). An Internet of Things-enabled model-based approach to improving the energy efficiency of aluminum die casting processes.

[37] Gao X. M., Yang S. F., Pan S. B. (2017). Optimal parameter selection for support vector machine based on artificial bee colony algorithm: a case study of grid-connected PV system power prediction. *Computational Intelligence and Neuroscience*.

[38] Ren S. Q., He K., Girshick R., Sun J. Faster R-CNN: towards real-time object detection with region proposal networks.

[39] Huang G. B., Zhu Q. Y., Siew C. K. (2006). Extreme learning machine: theory and applications. *Neurocomputing*.

[40] Zhuang F. Z., Qi Z., Duan K. (2021). A comprehensive survey on transfer learning. *Proceedings of the IEEE*.

[41] Zhou Q., Zhou W., Wang S. (2021). Semantic adaptation network for unsupervised domain adaptation. *Neurocomputing*.

[42] Jin Z., Kim J., Yeo H., Choi S. (2022). Transformer-based map-matching model with limited labeled data using transfer-learning approach. *Transportation Research Part C: Emerging Technologies*.

[43] Vecchietti A., Lee S., Grossmann I. E. (2003). Modeling of discrete/continuous optimization problems: characterization and formulation of disjunctions and their relaxations. *Computers & Chemical Engineering*.

[44] globalpetrolprices (2022). Retail energy price data. https://www.globalpetrolprices.com/.

[45] Lee N., Nee C. H., Yap S. S. (2022). Capacity sizing of embedded control battery–supercapacitor hybrid energy storage system. *Energies*.

